# *Bacillus* Phage vB_BtS_B83 Previously Designated as a Plasmid May Represent a New *Siphoviridae* Genus

**DOI:** 10.3390/v11070624

**Published:** 2019-07-07

**Authors:** Emma G. Piligrimova, Olesya A. Kazantseva, Nikita A. Nikulin, Andrey M. Shadrin

**Affiliations:** 1 Laboratory of Bacteriophage Biology, Skryabin Institute of Biochemistry and Physiology of Microorganisms, RAS, 142290 Pushchino, Russia; 2 Department of Microbiology, Federal State Budgetary Educational Institution of Higher Education “Vyatka State University”, 610000 Kirov, Russia

**Keywords:** bacteriophage, phage, *Bacillus thuringiensis*, plasmid prophage, *Bembunaquatrovirus*, *Siphoviridae*

## Abstract

The *Bacillus cereus* group of bacteria includes, inter alia, the species known to be associated with human diseases and food poisoning. Here, we describe the *Bacillus* phage vB_BtS_B83 (abbreviated as B83) infecting the species of this group. Transmission electron microscopy (TEM) micrographs indicate that B83 belongs to the *Siphoviridae* family. B83 is a temperate phage using an arbitrium system for the regulation of the lysis–lysogeny switch, and is probably capable of forming a circular plasmid prophage. Comparative analysis shows that it has been previously sequenced, but was mistaken for a plasmid. B83 shares common genome organization and >46% of proteins with other the *Bacillus* phage, BMBtp14. Phylograms constructed using large terminase subunits and a pan-genome presence–absence matrix show that these phages form a clade distinct from the closest viruses. Based on the above, we propose the creation of a new genus named *Bembunaquatrovirus* that includes B83 and BMBtp14.

## 1. Introduction

Over the past two decades, the number of bacteriophage genomes deposited in public databases has substantially increased due to the advances in sequencing technologies, which has led to an increasing interest in the field of phage taxonomy [1]. The first classification of tailed bacteriophages (phages) was proposed in the 1970s based on morphology and nucleic acid composition [2,3,4]. In subsequent years, new approaches appeared in phage taxonomy, including whole genome and proteome comparisons, based on which new phage taxa were established, including the *Ackermannviridae* and *Herelleviridae* families, as well as 19 subfamilies and more than 250 genera within *Myoviridae, Siphoviridae*, and *Podoviridae*.

The *Bacillus cereus* group of bacteria encompasses an increasing number of species, some of which are of medical and agricultural importance, including the best known and studied species *B. cereus, B. thuringiensis*, and *B. anthracis*. They are Gram-positive endospore-forming bacteria that are ubiquitous in many environments. The tailed phages of these bacteria are numerous and widespread, similar to their hosts, and are represented by many genera of the *Myoviridae, Siphoviridae*, *Herelleviridae*, and *Podoviridae* families, as well as by numerous unclassified phages. In addition to diverse morphology, they possess impressively diverse features such as lifestyles and lysogenic states: virulent phages, as well as temperate phages integrating into chromosomes, plasmids, or acting as independently replicating circular or linear plasmids, were reported for this group of bacteria [5]. The temperate phages of *Bacillus*, including those of the *Bacillus cereus* group members, were the first viruses where the “arbitrium” signal system similar to the quorum-sensing systems of their hosts was detected [6,7]. These facts indicate that *Bacillus* phages are full of surprises and, despite the large number of already sequenced genomes, they need to be carefully studied.

In this study, we describe the newly isolated temperate *Bacillus* phage vB_BtS_B83 (abbreviated as B83). The analysis of its genome has shown that it was previously deposited in GenBank but erroneously annotated as a plasmid. In addition, B83 possesses interesting features, such as the predicted extrachromosomal replication ability, and may be a member of a new phage genus along with another *Bacillus* phage BMBtp14. Based on our results, we propose the formation of the bacteriophage genus “*Bembunaquatrovirus*” to formally classify these phages.

## 2. Materials and Methods

### 2.1. Bacterial Strains and Growth Conditions

The bacterial strains used in this study were obtained from the All-Russian Collection of Microorganisms (VKM) and are listed in Table S1. Lysogeny broth (LB) and LB agar (1.5% *w*/*v* and 0.75% *w*/*v*) with 10 mM of CaCl_2_ and 10 mM of MgCl_2_ were used for bacterial and phage cultivation. All cultures were grown at 28 °C.

### 2.2. Phage Isolation and Propagation

Phage B83 was isolated from its host strain *B. thuringiensis* VKM B-83 by mitomycin C induction, as described by Moumen et al. [8]. Briefly, the strain was grown in 30 mL of LB broth with 10 mM of CaCl_2_ and 10 mМ of MgCl_2_ to the optical density of 0.3 at 600 nm. Then, mitomycin C was added to a final concentration of 0.2 μg/mL. The culture was incubated for 2 h at 28 °C until optical density decreased; then, 300 μL of chloroform was added. Then, the cell debris was removed by centrifugation at 12,800× *g* for 10 min. The obtained lysate was titrated with serial dilutions. Then, separate plaque was extracted with SM+ buffer (50 mM of Tris-HCl, pH 8.0; 100 mM of NaCl; 1 mM of MgSO_4_; 0.1% gelatin; 10 mM of CaCl_2_; 10 mM of MgCl_2_), and extraction–titration cycles were repeated five times in order to avoid contamination with other bacteriophages.

The sensitive strain *B. cereus* VKM B-370 was used for phage propagation to obtain high-titer suspension. Briefly, 300 μL of the overnight bacterial culture was transferred into 30 mL of LB with 10 mM of CaCl_2_ and 10 mM of MgCl_2_; then, 20 μL of phage extract was added. Incubation of the culture was carried out at 28 °C with a periodic monitoring of optical density. After the lysis, 1.8 g of NaCl and 300 μL of chloroform were added, and the incubation was continued for 30 min. Then, the cell debris was removed by centrifugation at 12,800× *g* for 10 min, and phages were precipitated from the supernatant with polyethylene glycol (PEG) 8000 (final concentration of 10%) and resuspended in 3 mL of SM+. The concentrated phage suspension was stored at 4°C.

### 2.3. Host Range Determination

Isolated phage B83 was characterized using a host range test against 30 strains of the *B. cereus* group. For this purpose, 5 μL of overnight bacterial cultures were transferred to test tubes with 1 mL of molten soft LB agar (0.75% *w*/*v* agar) with 10 mM of CaCl_2_ and 10 mM of MgCl_2_, which were mixed and overlaid on LB agar (1.5% *w*/*v*) in Petri dishes (60 mm in diameter). Next, 2 μL of a high-titer phage suspension (≥10^8^ plaque-forming units (PFU)/mL) were dripped onto the agar plates with the host strains. Incubation was performed for 24 h at 28 °C.

### 2.4. Transmission Electron Microscopy

Prior to electron microscopy, phages were purified by gel filtration on Sepharose 4B (GE Healthcare). Phage suspension applied onto 200 mesh formvar-coated copper grids was negatively stained with 2% uranyl acetate and subsequently analyzed using a JEM-2100 (JEOL Ltd., Tokyo, Japan) transmission electron microscope.

### 2.5. Phage DNA Sequencing and Analyzing

Bacteriophage DNA was sequenced using Illumina with TruSeq library preparation technology. The genomic sequence was assembled de novo using SPAdes v.3.11.1 software [9]. PAUSE3 [10] was used for searching genome ends. Open reading frames (ORFs) were identified with RASTtk [11] and subsequently analyzed manually. The putative functions of ORFs were predicted using BLAST (NCBI) [12,13] and HHpred [14]. ARAGORN [15] and tRNAscan-SE [16] were used for tRNA gene searching. The circular genome map was visualized with CGView [17].

### 2.6. Comparative Genomics

BLASTN was used to find genomes related to B83, and similarity was visualized with Easyfig [18]. Shared proteins were determined within the related genomes with CoreGenes 3.5 [19] and searched against all hidden Markov model (HMM) profiles downloaded from the prokaryotic Virus Orthologous Groups (pVOGs) database [20] using hmmscan from HMMer3.1 software [21]. Only the matches with an *E*-value of ≤1 × 10^−15^ and coverage of ≥35% of the profile HMM were considered significant. Homologous gene clusters from the predicted proteomes were computed using GET_HOMOLOGUES software [22] with the COGtriangles algorithm [23,24] (with a threshold of 75% for query coverage and 1 × 10^−5^ for *E*-value on the all-against-all BLASTP results). A homologous gene cluster presence/absence matrix was created with compare_clusters.pl script from the GET_HOMOLOGUES package, excluding the clusters with inparalogs. A parsimonious tree was built using GET_PHYLOMARKERS [25] with parameters -R 3, -b 1000, -j 10. A large terminase subunit phylogram was generated in MEGAX [26], applying MUSCLE for sequence alignment [27] and the maximum likelihood (ML) method with the Whelan and Goldman substitution model [28] for tree inference, with 1000 bootstrap replicates. FigTree v1.4.4 was used for visualization of the trees [29].

### 2.7. Accession Number

The genome sequence of phage B83 was submitted to GenBank under accession number MK759918.1.

## 3. Results

### 3.1. Isolation, Host Range and Morphology

*Bacillus* phage В83 was isolated by mitomycin C induction from its host strain *B. thuringiensis* VKM B-83, which has a high level of similarity to the *B. thuringiensis* subsp. *israelensis* ATCC35646 according to fingerprint analysis [30]. The isolated phage was tested against a collection of 30 strains of the *B. cereus* group for host range determination. B83 lysed three (10%) out of these strains, including *B. cereus* VKM B-370, VKM B-373, and VKM B-473 (Table 1). On the propagating host strain VKM B-370, B83 produced turbid plaques with an approximate diameter of 1 mm (Supplementary Information, Figure S1).

**Figure 1:**
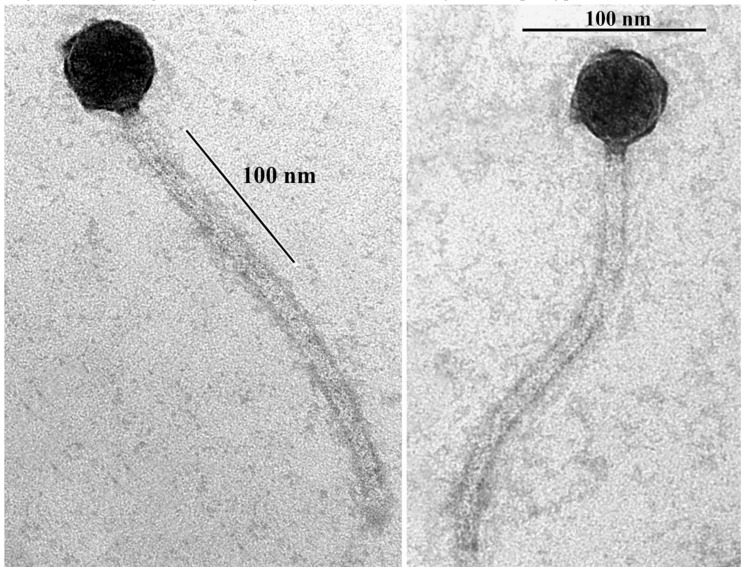
Transmission electron microscopy of *Bacillus* phage B83 negatively stained with 2% (*w*/*v*) uranyl acetate. Scale bars represent 100 nm.

As determined by transmission electron microscopy, B83 has a non-elongated capsid (approximately 45 nm in diameter) and a flexible noncontractile tail (approximately 240 nm in length) (Figure 1), indicating that it belongs to the *Siphoviridae* family and morphotype B1 [31].

### 3.2. General Genome Organization of B83

**Figure 2:**
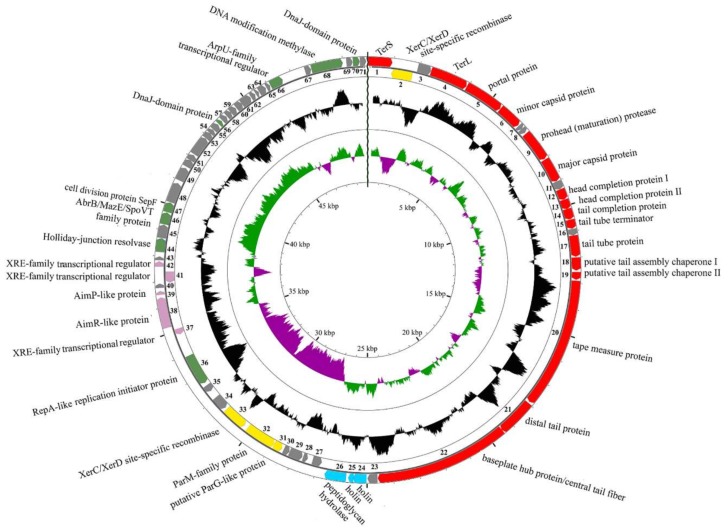
The B83 genome map. Red arrows – open reading frames (ORFs) with predicted morphogenesis functions, cyan arrows – lytic functions, yellow arrows – plasmid maintenance, pink arrows – lysis-lysogeny switch, green arrows – DNA replication, recombination and modification. Gray arrows indicate ORFs with unknown functions. Black middle diagram shows GC-content, and internal purple-green diagram shows GC-skew.

The resulted reads were successfully assembled into a single contig with the average coverage of 256. The genome contains 49,952 bp with GC-content 35.8% and 71 predicted ORFs. We were able to assign the putative functions of 36 ORFs using BLAST search and HHpred as an accessory method. The genome map is shown in Figure 2 with the first base of the putative terminase small subunit gene as the first base of the genome. The more detailed description of predicted ORFs is given in Table S2.

We have identified the structural, lytic, and DNA metabolism genes encoded on one strand and a set of genes scattered around the genome and encoded on the opposite strand. The latter contains 13 ORFs; some of them seem to be closely related to plasmid-encoded genes. Among them, we were able to predict XerC/XerD-family site-specific recombinases (ORFs 2 and 33) with ORF2 more similar to XerD and ORF33 to XerC (Table S2), ParM-family protein (ORF 32), and replication initiator protein similar to plasmid replication initiator RepA (ORF 36).

**Figure 3:**
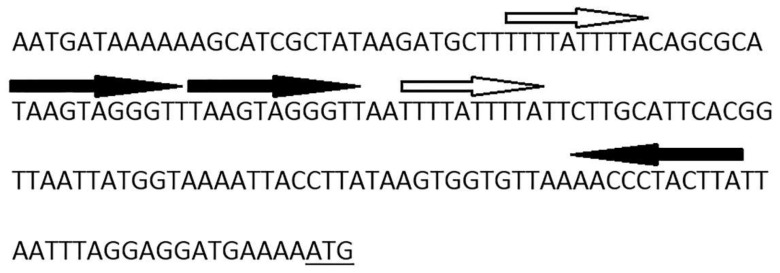
Nucleotide sequence of the region upstream of ORF32 showing the iterated sequences. The ORF32 start codon is underlined.

It is not so typical for replication initiator genes to be transcribed in the same orientation with site-specific recombinase genes in temperate phages [32] usually relying on host replication machineries in the prophage state, so we have assumed that B83 can use its own RepA-like initiator to replicate extrachromosomally during the lysogenic cycle, as it was described for some other phages, e.g., *Escherichia* phage P1 [33]. The presence of ORF32 with a high amino acid sequence similarity to bacterial plasmid protein ParM is known to be required for successful plasmid segregation [34], and also suggests that B83 may exist as a circular plasmid in host cytoplasm. ParM is an actin-like motor protein of the ParMRC segregation system, which forms long filaments pushing plasmids to opposite poles of the cell to prevent their loss during cell division [35]. The ParMRC system has other two components: a centromere-like iterons-containing DNA region *parC* and a small DNA-binding protein ParR transcribed immediately downstream the *parM* gene, which serves as an adaptor between ParM and *parC* [34]. Interestingly, the product of B83 ORF31 exhibits no homology to ParR, but contains the predicted N-terminal ribbon–helix–helix fold similar to that of the members of the MetJ/Arc-superfamily of DNA-binding proteins and ParG. ParG is an adaptor protein of another plasmid segregation system, and it functions generally similar to ParR, binding DNA in the iterons-containing region [36,37]. We have identified iterons within the 165-bp region preceding ORF32, which may be the binding site for the ParG-like adaptor protein (Figure 3), so in this case, B83 seems to have all of the three essential elements of a plasmid segregation system.

To confirm that B83 can exist as the plasmid in the lysogenized cells, we compared its restriction patterns with those of the plasmid DNA extracted from the host lysogen *B. thuringiensis* VKM B-83. The resultant profiles indicate that phage DNA is indeed presented in plasmid DNA fraction in host cells in an amount that is comparable with other plasmid DNA (Figure S2). Nevertheless, we cannot exclude that in some cases, the phage DNA can be integrated into the host chromosome.

ORF37, 41, and 42 products are XRE-family transcriptional regulators that are similar to lambda CI, CII, and Cro repressors (Table S2), indicating that these genes are involved in lysis-lysogeny switch regulation. Three genes located between them are components of the arbitrium system discovered in 2017 by Erez et al. in *Bacillus* phages phi3T and SPbeta [6]. Arbitrium systems are widespread among *Bacillus*-infecting temperate phages and include AimP, which is a small peptide playing the role of a signal molecule, AimX, which is a non-coding RNA that is a negative regulator of lysogeny, and AimR, which is the cellular receptor of AimP and transcriptional activator of AimX [6]. ORF38 of B83 encodes a product with predicted structural similarity to the AimR protein (Table S2), and the product of the next ORF39 is an AimP-like 46-amino acid protein with the presence of a predicted signal peptide cleavage site between positions 23–24, so that the sequence of the generated pro-peptide is EKASKQESNKQVYYMADPGGSVG. ORF40 may be a functional homolog of small ORFs with currently unknown functions, which are often present in AimX transcripts (as in case of phage phi3T but not SPbeta) [6].

As it has been reported by A. Stokar-Avihail et al. in a recent work, all variety of the known arbitrium systems may be divided into nine clades based on the phylogeny of AimR homologs, where each clade has its own characteristic profile of mature AimP sequence [7]. Multiple alignments of the AimR-like protein from B83 with these reported AimR homologs has shown that it is most similar to the proteins from clade 7 (up to 98% of BLASTP identity), which includes AimR homologs from prophages, phages, and conjugative elements of *B. wiedmannii*, *B. cereus*, *B. anthracis*, *B. weihenstephanensis*, *B. mycoides*, and *B. thuringiensis* [7], with some of them having identical sequences of AimP-like proteins with B83.

The DNA packaging genes and typical *Siphoviridae* morphogenesis genes of B83 were identified on the basis of sequence homology (BLAST) and predicted structural similarity (HHpred) with resolved structures of the known orthologous proteins, considering the conservative gene order in structural modules [38]. These genes, which are described in more detail in Table S2, include small and large terminase subunits (ORF1 and ORF4, respectively), head portal protein (ORF5), minor capsid protein (ORF6), prohead protease (ORF9), major capsid protein (ORF10), YqbG-like and XkdH-like head completion proteins (ORFs 12 and 13, respectively), tail completion protein (ORF14), tail tube terminator (ORF15), tail tube protein (ORF17), two putative tail assembly chaperones (ORFs 18 and 19), tape measure protein (ORF20), distal tail protein (ORF21), and baseplate hub protein encoded within the same ORF with the central tail fiber protein (ORF22), which is a common feature of Gram-positive infecting siphophages [38].

B83 lytic genes include two holins, each with two transmembrane regions (ORFs 24 and 25), and a peptidoglycan hydrolase with predicted *N*-acetylmuramoyl-l-alanine amidase activity (ORF26).

### 3.3. Comparative Genomics

BLASTN search with B83 whole genome sequence revealed three related genomes. One was sequenced by Cao et al. as a part of work on the melanin-producing strain *B. thuringiensis* L-7601 [39], and is completely identical to B83 (total nucleotide identity of 100%, calculated as "Ident" multiplied by "Coverage" values), although designated as a plasmid (plasmid unnamed 2, GenBank accession number CP020004.1). The second genome, which is also marked as a plasmid (pBTHD521-2, GenBank accession number CP010108.1), was obtained by Li et al. from *B. thuringiensis* HD521 [40], and has a total nucleotide identity of 64.7% to B83. A single related genome designated as a phage is vB_BtS_BMBtp14, with a total nucleotide identity of 37%. A BLASTN search against the Viruses database (taxon:10239) revealed no phage genomes with total nucleotide identity more than 10% except for BMBtp14. Thus, we have assumed that B83, along with BMBtp14 and the related phages mistakenly annotated as plasmids, may represent a new *Siphoviridae* genus with *B. thuringiensis* L-7601 plasmid 2 belonging to the same species with B83 (as the currently used species demarcation criterion is the genome nucleotide identity of 95% [1]).

To determine a number of shared proteins encoded in the four genomes, we performed an online CoreGenes3.5 analysis with threshold 200, and manually verified the results. As is presented in Table 2, the portion of homologous proteins is significant, despite various annotation methods. A total of 33 proteins are common to all four genomes, i.e., from 44% to 53.2%. These values exceed the threshold of >40% of shared proteins previously used to classify phages into one genus [41,42,43]. We have affiliated 15 out of these 33 proteins to the known pVOGs (Table 3). 

**Figure 4:**
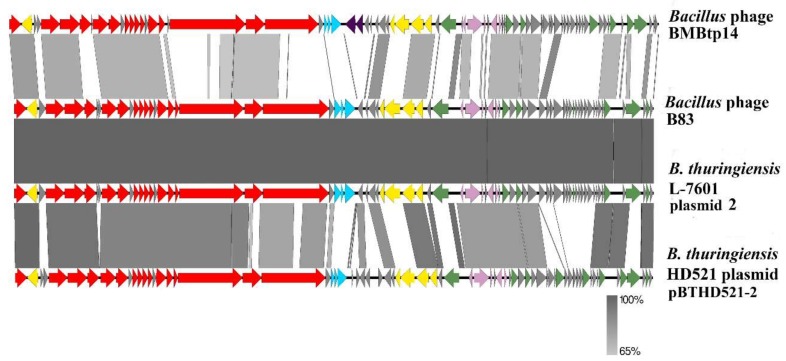
The BLASTN comparison of the B83 genome and the related genomes visualized with Easyfig. Red arrows – ORFs with predicted morphogenesis functions, cyan arrows – lytic functions, yellow arrows – prophage maintenance, pink arrows – lysis-lysogeny switch, green arrows – DNA replication, recombination, and modification. Dark purple arrows in the BMBtp14 genome indicate the genes absent in the other three genomes, which are probably associated with the mobile genetic element. Gray arrows indicate ORFs with unknown functions. Gray regions between the genome maps indicate the level of identity (see the legend).

The genomes of BMBtp14, *B. thuringiensis* L-7601 plasmid unnamed 2, and *B. thuringiensis* HD521 plasmid pBTHD521-2 were downloaded from GenBank, and ORFs were re-searched using RASTtk. Then, the genomes were manually colinearized, placing the starting points at the first codons of the small terminase genes, and pairwise nucleotide identity was visualized with Easyfig (Figure 4). The high level of similarity in structural modules and whole-genome synteny confirm that “plasmid” genomes belong to related siphoviruses. All these phages possess plasmid maintenance genes and arbitrium systems similar to those of B83, indicating that they are temperate and form circular extrachromosomal prophages, which most likely caused erroneous annotation.

In order to confirm our assumption that B83 and three related phages form a separate group genetically distant from other described viruses, we alternately searched for all B83 translated ORFs using BLASTP against NCBI Viruses database (taxon:10239). A set of 105 phage genomes with evident homologous proteins was collected, including phages from the *Siphoviridae*, *Myoviridae*, and *Herelleviridae* families (Table S3) and re-annotated genomes of BMBtp14, *B. thuringiensis* L-7601 plasmid unnamed 2, and *B. thuringiensis* HD521 plasmid pBTHD521-2. Then, homologous gene families from the predicted proteomes of these viruses and B83 were computed using GET_HOMOLOGUES software. All the genes were grouped into 5217 clusters, with no cluster containing genes from all the phages.

**Figure 5:**
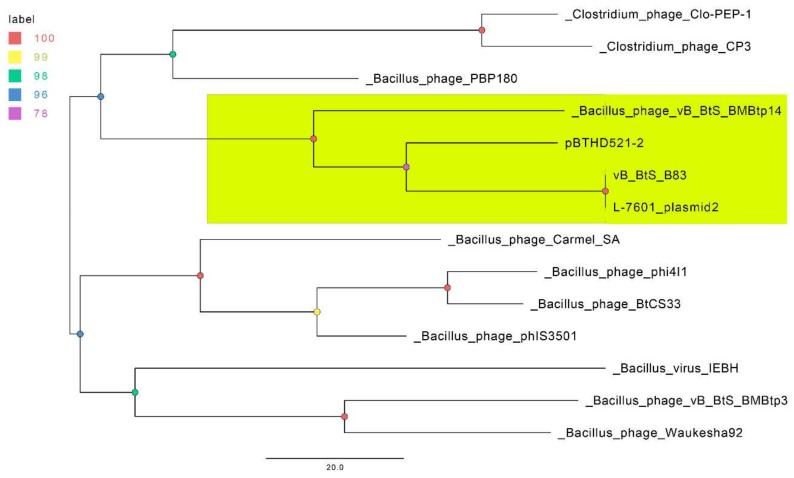
The parsimony pan-genome tree for 14 phage genomes derived from the presence/absence of homologous genes in a pan-genome matrix. The nodes are colored according to the legend, which represents standard bootstrap support values. The clade containing the members of proposed new genus is highlighted in yellow.

Due to the absence of detected core and soft-core genomes within this set of phages, we reduced it to 13 phages with the largest numbers of genes clustered together with the B83 genes (four to 71). This set was used for constructing a parsimonious tree and included *B. thuringiensis* L-7610 plasmid unnamed 2, *B. thuringiensis* HD521 plasmid pBTHD521-2, *Bacillus* phages BMBtp14, BMBtp3, IEBH, phIS3501, PBP180, Carmel_SA, phi4I1, Waukesha92, BtCS33, and *Clostridium* phages Cp3 and Clo-PEP-1, which are listed in Table S4 together with the homologous proteins. The resultant tree (Figure 5) shows that B83 and the three related genomes form a clade distinct from the closest phages, with B83 and *B. thuringiensis* L-7601 plasmid unnamed 2 apparently representing the same phage species.

**Figure 6:**
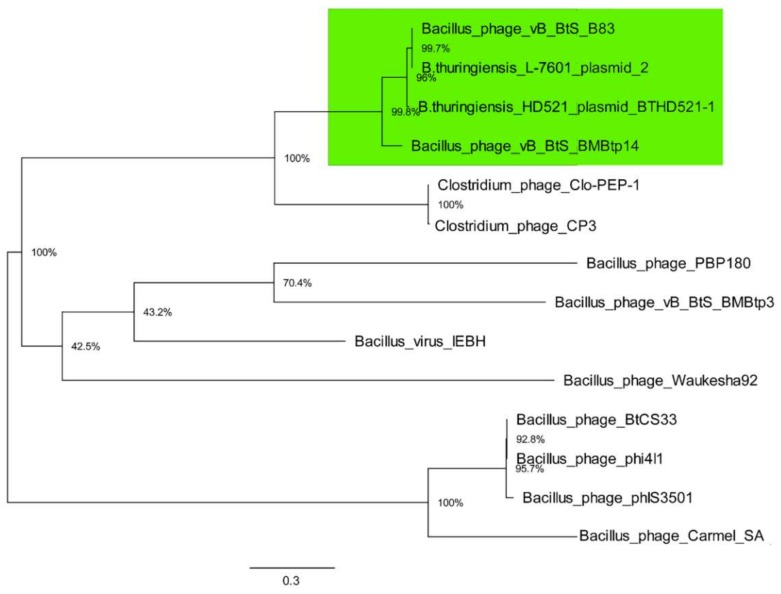
Phylogenetic analysis of amino acid sequences of TerL of 14 phages using maximum likelihood (Whelan and Goldman substitution model), with 1000 bootstrap replicates. Scale bar represents the number of amino acid substitutions per site. The clade containing the members of the proposed new genus is highlighted in green.

Then, the phylogenetic analysis of TerL amino acid sequences from the same 14 genomes with maximum likelihood was performed, which is a standard phage phylogenetic comparison method. The resultant tree (Figure 6) shows that TerL proteins from B83, BMBtp14, plasmid pBTHD521-2, and plasmid unnamed 2 form a clade, which is consistent with the pan-genomic tree (Figure 5), indicating that these phages represent a distinct branch of viruses. As long as two members of this clade were mistaken for plasmids, we propose to create a new *Siphoviridae* genus including only *Bacillus* phages BMBtp14 and B83, which we have tentatively named “*Bembunaquatrovirus*” based on the first sequenced member (BMBtp14). On average, the genomes of members of this genus are 50.4 kb with a GC-content of 36.3%, 73 ORFs, and no tRNA genes. They have a relatively low whole-genome nucleotide identity (BLASTN) of approximately 37%, but a high level of genomic collinearity and share 35 proteins, as determined with CoreGenes3.5, which is from 46.7% to 49.3% and exceeds a threshold of >40%, which was previously used for the definition of phage genera [41,42,43].

### 3.4. Determination of Packaging Strategy

**Figure 7:**
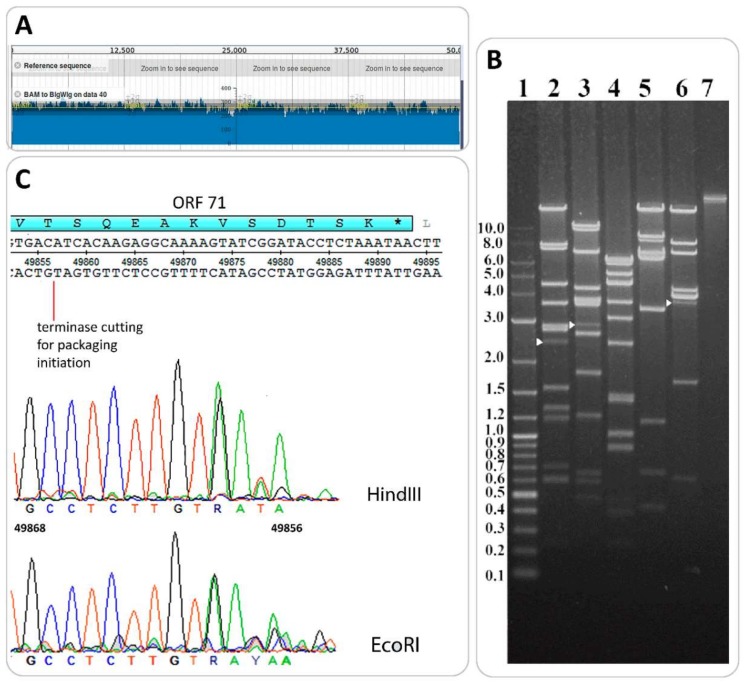
Determination of packaging strategy. (**A**). Genome coverage plot was obtained with PAUSE3 [10]. No peaks deviate significantly from the mean (yellow line). (**B**). Restriction analysis of B83 DNA with enzymes HindIII (lane 2), EcoRI (lane 3), NsiI (lane 4), XbaI (lane 5), and BsrGI (lane 6). Molecular weight markers and intact DNA are in lanes 1 and 7, respectively. White arrows indicate *pac*-fragments. (**C**). Location of terminase-generated cut in the B83 genome. Terminal regions of sequencing reads for HindIII-generated and EcoRI-generated *pac*-fragments are displayed in sequencing chromatograms.

The putative end positions in the B83 genome were predicted with PAUSE3 (Pile-up Analysis Using Starts and Ends) [10] by mapping individual reads on the genomic sequence. Relatively homogeneous coverage with no significant peaks (with double or more than double depth of coverage) was observed on the coverage plot (Figure 7A), which is typical of phages with circularly permuted terminal repeats using the headful mechanism of DNA packaging [44].

The direct experimental validation of headful packaging was performed by the restriction digest of B83 DNA with restriction endonucleases HindIII, EcoRI, NsiI, XbaI, and BsrGI. In the cases of enzymes HindIII, EcoRI, and BsrGI, we were able to see submolar fragments (*pac*-fragments) with the lengths of 2.5 kb, 2.9 kb, and 3.5 kb, respectively, appearing on the tracks in addition to all the predicted restriction fragments (Figure 7B). This type of restriction pattern is considered to be diagnostic of headful packaging [31]. The DNA of *pac*-fragments was extracted from electrophoresis gel and used for Sanger sequencing with primer 5’-TGTTAATAGGAGCGCTCTCT-3’, annealing 200 bp downstream the first codon of the small terminase subunit gene, given the conserved location of the *pac*-site within or near this gene in headful packaging phage genomes [44]. For HindIII-generated and EcoRI-generated *pac*-fragments, we were able to obtain high-quality reads mapping a terminase-generated cut approximately in position 49857 of the genome, within ORF71, as is shown in sequencing chromatograms (Figure 7C). Thus, B83 apparently uses the headful mechanism of DNA packaging with accurate site-specific initiation. 

## 4. Discussion

The temperate *Bacillus* phage B83 that was isolated and characterized in this work belongs to a distinct group of viruses along with *Bacillus* phage BMBtp14 and two other *Bacillus* phages designated as plasmids in GenBank (*B. thuringiensis* L-7601 plasmid unnamed 2 and *B. thuringiensis* HD521 plasmid pBTHD521-2). Comparative analyses have revealed that the nucleotide genomic sequence of B83 is almost identical to those of *B. thuringiensis* L-7601 plasmid unnamed 2 (Table 2, Figure 4) and, therefore, they represent the same phage species. Another two phages exhibit pairwise nucleotide identity of ≥37% (BLASTN) and shared protein content of ≥46% (CoreGenes) with B83. They also form a clade together with B83 in the pan-genome (Figure 5) and TerL (Figure 6) phylograms when compared to a set of genetically closest phages selected by BLASTP homology with B83 proteins. This is consistent with the results of homologous gene clustering (Table S4), indicating that only four to six homologous genes are contained in the other closest phage genomes. Whole-genome BLASTN comparison against the Viruses database also shows that there are no described phages with ≥10% of total nucleotide identity to B83, except for BMBtp14.

B83, BMBtp14, and pBTHD521-2 have highly similar whole-genome organization (Figure 4), with the most of the genes encoded on one strand and 18% to 23% of the genes encoded on the opposite strand. The latter includes the genes that are known to play a role in plasmid replication, such as XerC/XerD site-specific recombinases and ParM-like protein involved in plasmid multimer resolving and partitioning [45,46,47]. The presence of the plasmid segregation proteins is characteristics of phages that were described as forming circular autonomously replicating plasmid prophages, such as *Bacillus* phages TP-21 [48], SU-11 [49], and IEBH [50], as well as Bp8p-C and Bp8p-T, for which the effect of ParM-like protein on lysogeny was experimentally confirmed [51]. This may indicate that B83, BMBtp14, and pBTHD521-2 are able to form circular plasmid prophages instead of integration into the host chromosome. When sequencing total bacterial DNA, such extrachromosomal prophages form circular contigs that can be mistaken for plasmids, but the presence of all the typical clusters of phage genes, such as morphogenesis, DNA packaging, and lysis genes, can reliably indicate the nature of the circular molecules. In this work, it was shown that B83 can exist as the circular plasmid in the cytoplasm of the host strain *B. thuringiensis* VKM B-83 (Figure S2). However, more detailed studies are needed for clear understanding of the lysogenic states of B83 and related phages.

The genes of AimR-like and AimP-like proteins of arbitrium systems were found in B83, BMBtp14, and pBTHD521-2 genomes surrounded by genes similar to lambda CI and Cro repressors, indicating that these phages have a sophisticated mechanism for lysis-lysogeny switch regulation. Their arbitrium systems belong to the clade 7; they are allocated on the basis of the phylogeny of AimR homologs by Stokar-Avihail et al. [7], and the sequences of mature AimP-like proteins currently need to be experimentally proven.

B83 was predicted to use the headful mode of DNA packaging, and the suggestion was experimentally verified (Figure 7). Since TerL homology usually correlates with the similarity of packaging mechanisms [31,44], it is clear that BMBtp14 and pBTHD521-2 displaying the TerL amino acid sequence identity to the B83 large terminase of 83.4% and 97.5%, respectively, are also headful packaging phages. Despite no micrographs being reported for these phages, they undoubtedly are the members of the *Siphoviridae* family because of the presence of structural genes that are typical of siphoviruses and highly similar to those of B83.

Based on our results, we propose the establishment of the genus “*Bembunaquatrovirus”* within the *Siphoviridae* family encompassing only two members, *Bacillus* phages B83 and BMBtp14, since the B. *thuringiensis* HD521 plasmid pBTHD521-2 was not annotated as a phage. The shared protein content of >40%, together with similar morphology, whole-genome collinearity, and conserved defining features were chosen as a criterion for genus definition, as it was previously applied for the classification of members of the *Siphoviridae, Myoviridae,* and *Podoviridae* families [41,42,43]. The genus name is derived from the first sequenced member, BMBtp14. We suggest that B83 be allocated as a type species, due to having a more detailed description. 

## Figures and Tables

**Table 1:** The host range of *Bacillus* phage В83 on 30 *Bacillus* strains.

No.	Species	Strain	Lysis *
1	*B. cereus*	VKM B-13	−
2	*B. cereus*	VKM B-370	+
3	*B. cereus*	VKM B-373	+
4	*B. cereus*	VKM B-383	−
5	*B. cereus*	VKM B-445	−
6	*B. cereus*	VKM B-473	+
7	*B. cereus*	VKM B-491	−
8	*B. cereus*	VKM B-504^T^	−
9	*B. cereus*	VKM B-682	−
10	*B. cereus*	VKM B-683	−
11	*B. cereus*	VKM B-684	−
12	*B. cereus*	VKM B-686	−
13	*B. cereus*	VKM B-688	−
14	*B. cereus*	VKM B-771	−
15	*B. cereus*	VKM B-810	−
16	*B. cereus*	VKM B-812	−
17	*B. cereus*	ATCC 4342	−
18	*B. cereus*	ATCC 14893	−
19	*B. thuringiensis*	VKM B-83	−
20	*B. thuringiensis*	VKM B-84	−
21	*B. thuringiensis*	VKM B-85	−
22	*B. thuringiensis*	VKM B-440	−
23	*B. thuringiensis*	VKM B-446	−
24	*B. thuringiensis*	VKM B-450	−
25	*B. thuringiensis*	VKM B-453	−
26	*B. thuringiensis*	VKM B-454	−
27	*B. thuringiensis*	VKM B-1555	−
28	*B. thuringiensis*	VKM B-1557	−
29	*B. thuringiensis*	ATCC 35646	−
30	*B. weihenstephanensis*	KBAB4	−

* Results recorded as +, lysis; −, no detectable lysis.

**Table 2:** The properties of B83 and the most closely related genomes.

Name	GenBank Accession No.	Genome Length (kb)	G+C-Content, %	ORFs	tRNA	Total DNA Sequence Identity *, %	Homologous Proteins **, % (Number)
B83	MK759918.1	49,952	35.79	71	0	100	100
plasmid unnamed 2	CP020004.1	49,952	35.79	62	0	100	100 (62)
plasmid pBTHD521-2	CP010108.1	49,838	35.91	70	0	64.7	60.0 (42)
BMBtp14	KX190833.1	50,740	36.76	75	0	37.0	46.7 (35)

* Determined using BLASTN compared to B83 (multiplying % coverage by % identity); ** Determined using CoreGenes3.5 compared to B83.

**Table 3:** Thirty-three conserved genes among the four genomes (B83, plasmid unnamed 2, pBTHD521-2, BMBtp14) determined by CoreGenes3.5, and their annotation (as was assigned for B83). Prokaryotic Virus Orthologous Groups (pVOGs) determined from translated open reading frames (ORFs) are presented.

No.	Product	B83 Protein id	B83 Locus Tag	pVOG
1	terminase small subunit	QCQ57781.1	B83_gp01	-
2	XerC/XerD site-specific recombinase	QCQ57782.1	B83_gp02	VOG0275
3	terminase large subunit	QCQ57785.1	B83_gp04	VOG4544
4	portal protein	QCQ57783.1	B83_gp05	VOG4773
5	putative phage head morphogenesis protein (minor capsid protein)	QCQ57786.1	B83_gp06	VOG4688
6	phage prohead (maturation) protease	QCQ57790.1	B83_gp09	VOG4685
7	major capsid protein	QCQ57789.1	B83_gp10	VOG0749
8	hypothetical protein	QCQ57791.1	B83_gp11	-
9	phage head completion protein (neck protein) I	QCQ57792.1	B83_gp12	-
10	phage head completion protein (neck protein) II	QCQ57793.1	B83_gp13	-
11	tail completion protein	QCQ57794.1	B83_gp14	-
12	tail tube terminator	QCQ57795.1	B83_gp15	-
13	hypothetical protein	QCQ57796.1	B83_gp16	-
14	tail tube protein	QCQ57797.1	B83_gp17	-
15	putative tail assembly chaperone	QCQ57798.1	B83_gp18	-
16	tail tape measure protein	QCQ57800.1	B83_gp20	-
17	distal tail protein	QCQ57801.1	B83_gp21	VOG4605
18	baseplate hub protein/central tail fiber	QCQ57802.1	B83_gp22	VOG4599
19	hypothetical protein	QCQ57809.1	B83_gp29	-
20	XerC/XerD site-specific recombinase	QCQ57813.1	B83_gp33	VOG0275
21	MerR family domain-containing protein	QCQ57835.1	B83_gp34	-
22	XRE family transcriptional regulator	QCQ57816.1	B83_gp37	-
23	AimR-like protein	QCQ57817.1	B83_gp38	VOG1563
24	XRE family transcriptionalregulator	QCQ57820.1	B83_gp41	-
25	Holliday junction resolvase	QCQ57823.1	B83_gp44	VOG5617
26	hypothetical protein	QCQ57824.1	B83_gp45	-
27	AbrB/MazE/SpoVT family DNA-binding domain-containing protein	QCQ57825.1	B83_gp46	VOG6628
28	cell division protein SepF	QCQ57826.1	B83_gp47	VOG9647
29	hypothetical protein	QCQ57827.1	B83_gp48	-
30	hypothetical protein	QCQ57844.1	B83_gp64	-
31	ArpU family transcriptional regulator	QCQ57846.1	B83_gp66	VOG0198
32	DNA methyltransferase	QCQ57848.1	B83_gp68	VOG4571
33	hypothetical protein	QCQ57849.1	B83_gp69	-

## Supplementary Materials

The following are available online at https://www.mdpi.com/1999-4915/11/7/624/s1, Supplementary Information, Table S1: Bacterial strains used in the study; Figure S1: *Bacillus* phage B83 plaque morphology on 0.75% w/v LB overlay; Table S2: Annotation of *Bacillus* phage B83; Figure S2: Phage DNA restriction patterns compared with those of the plasmid DNA purified by alkaline lysis method from the host lysogen *B. thuringiensis* VKM B-83; Table S3: Phage genomes used for homologous gene clustering; Table S4: Phage genomes used for phylogenetic inference.
