# Supplementary material for: Bacillus Phage vB_BtS_B83 Previously Designated as a Plasmid May Represent a New Siphoviridae Genus
Source: Viruses. 2019 Jul 7;11(7):624. doi: 10.3390/v11070624 (PMC6669507; doi:10.3390/v11070624)
Supplement: Supplementary file 1 [file viruses-11-00624-s001.pdf]

Table S1: Bacterial strains used in the study

№	Species	Strain
1	*B. cereus*	VKM B-13
2	*B. cereus*	VKM B-370
3	*B. cereus*	VKM B-373
4	*B. cereus*	VKM B-383
5	*B. cereus*	VKM B-445
6	*B. cereus*	VKM B-473
7	*B. cereus*	VKM B-491
8	*B. cereus*	VKM B-504^T
9	*B. cereus*	VKM B-682
10	*B. cereus*	VKM B-683
11	*B. cereus*	VKM B-684
12	*B. cereus*	VKM B-686
13	*B. cereus*	VKM B-688
14	*B. cereus*	VKM B-771
15	*B. cereus*	VKM B-810
16	*B. cereus*	VKM B-812
17	*B. cereus*	ATCC 4342
18	*B. cereus*	ATCC 14893
19	*B. thuringiensis*	VKM B-83
20	*B. thuringiensis*	VKM B-84
21	*B. thuringiensis*	VKM B-85
22	*B. thuringiensis*	VKM B-440
23	*B. thuringiensis*	VKM B-446
24	*B. thuringiensis*	VKM B-450
25	*B. thuringiensis*	VKM B-453
26	*B. thuringiensis*	VKM B-454
27	*B. thuringiensis*	VKM B-1555
28	*B. thuringiensis*	VKM B-1557
29	*B. thuringiensis*	ATCC 35646
30	*B. weihenstephanensis*	KBAB4

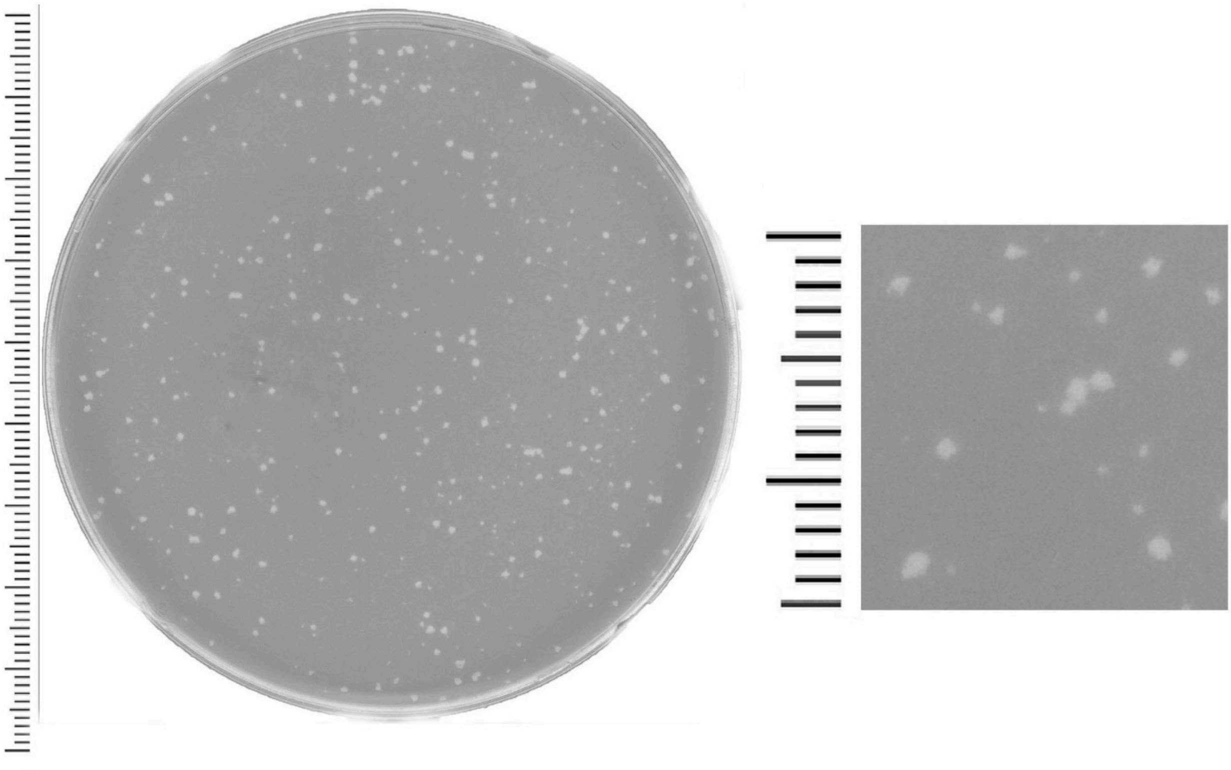

Figure S1: *Bacillus* phage B83 plaque morphology on 0.75% w/v LB overlay.

Table S2. Annotation of *Bacillus* phage B83

**ORF №**	**Position in genome**		**BlastP results**		**Conserved domains, BlastP**		**HHpred results (Prob./E-val)**	**Predicted function**
	**Start /stop codons**	**Strand**	**Name**	**e-val**	**Name, (region)**	**E-val**		
1	1/942	+	TerS protein	$2e^{-41}$	YjcR (1-771)	$2.62e^{-19}$		Phage terminase, small subunit
					Phage_terminase (1-165)	$2.32e^{-6}$		
2	1783/947	-	Integrase	0.0	XerC (3-251)	$2.11e^{-57}$		XerC/XerD site-specific recombinase
					recomb_XerC (5-261)	$5.28e^{-55}$		
					XerD (1-251)	$1.53e^{-50}$		
					INT_XerDC_C (108-251)	$7.29e^{-42}$		
					Phage_integrase (106-263)	$1.35e^{-37}$		
3	1937/2452	+	hp				1) PF10593.9 – 81.08/13<br>2) Acetyltransferase [*S. aureus*] – 74.04/40	hp
4	2459/3922	+	Terminase B [*Bacillus thuringiensis*]	0.0				Phage terminase, large subunit
5	3939/5465	+		e^{-77}	COG5518	$3.27e^{-144}$		

			Phage-like element PBSX portal protein xkdE		portal_PBSX	$4.22e^{-77}$		Phage XkdE-like portal protein
6	5481/6401	+	Phage head morphogenesis protein	$6e^{-73}$			1) COG2369 (Mu gpF-like) 99.8/$6.7e^{-20}$ 2) TIGR01644 (phage_SPP1_gp7) 99.77/$4.5e^{-21}$ 3) PF06152.11 (phage_min_caps2) 99.73/$1.6e^{-18}$	Putative phage head morphogenesis protein (minor capsid protein)
7	6436/6600	+	hp				1) PF10122.9 (Mu-like_Com) 95.88/0.0049	hp
8	6597/6785	+	hp					hp
9	6837/8033	+	Terminase	$4e^{-125}$	Peptidase_S78_2 (130-459)	$3.11e^{-39}$	1) PF14550.6 Peptidase_S78_2 Putative phage serine protease XkdF 99.88/$9.2e^{-25}$	Phage prohead (maturation) XkdF-like serine protease
			Phage portal protein	$1e^{-105}$				
10	8061/8996	+	Phage major capsid protein	0.0	Phage_capsid (27-306)	$1.69\ e^{-12}$		Phage major casid protein
					maior_cap_HK97 (12-193)	$5.65e^{-5}$		
11	9008/9331	+	hp		YqbF (7-47)	$2.6e^{-11}$	1) 2HJQ_A (hp yqbF) 100/$3.8e^{-43}$	hp

							2) 2OUT_A (Mu-like prophage FluMu gp35) 82.79/3	
12	9337/9753	+	DUF3199 family protein	$1e^{-96}$	DUF3199 (3-136)	$2.71e^{-32}$	1) d1xn8a_ (hp YqbG – *B. subtilis*) $99.84/2.1e^{-22}$	Phage head completion protein (neck protein) I (Yqbg-like)
			Protein YqbG (plasmid)	$3e^{-75}$	Yqbg (4-122)	$5.6e^{-21}$		
13	9756/10130	+	hp				1) Phage-like element PBSX protein xkdH; $99.69/1.1e^{-18}$	Phage head completion protein (neck protein) II (XkdH-like)
14	10130/10543	+	hp				1) TIGR01725 (phage_HK97_gp10) 96.46/0.0025<br>2) PF04883.12 (HK97-gp10_like) 96.28/0.0023<br>3) COG5005 (Mu-like prophage protein gpG) 91.51/0.95	Phage tail completion protein
15	10554/10904	+	hp				1) PF06141.11 (Phage_tail_U) 91.58/4	Tail tube terminator protein (gpU-like)
16	10919/11167	+	hp					hp
17	11167/11979	+	hp				1) TIGR01603 (maj_tail_phi13) 87.77/6.7	Phage tail tube protein (major tail protein)

							2) PF04630.12 (Phage_TTP_1) 65.15/140	
18	12012/12506	+	hp				PF10109.9 (Phage_TAC_7) 26.42/410	Putative phage tail assembly chaperone
19	12569/12859	+	hp				PF10666.9 (Phage_TAC_8) 23.11/450	Putative phage tail assembly chaperone
20	12899/17926	+	Phage tail tape measure protein	0.0	tape_meas_TP901 (250-660)<br>PhageMin_Tail (289-553)<br>COG5283 (110-813)<br>DUF4455 (732-1059)<br>TIGR02680 (43-216)<br>COG5290 (762-935)<br>PTZ00121 (742-1102)	$5.05e^{-37}$<br>$1.35e^{-30}$<br>$4.76e^{-06}$<br>$4.39e^{-4}$<br>$1.73e^{-3}$<br>$2.04e^{-3}$<br>$4.45e^{-3}$		Phage tail tape measure protein
21	17985/19433	+	Phage tail protein<br>Phage tail fiber protein [*Bacillus* phage Gamma isolate d'Herelle]<br>Distal tail protein [*Bacillus* phage phi4J1]	0.0<br>0.0<br>$5e^{-150}$	Sipho_tail (6-176)<br>phi3626_gp14_N (4-112)<br>YomH (3-147)<br>Sipho_tail (409-481)<br>YomH (420-480)	$1.55e^{-38}$<br>$2e^{-27}$<br>$8.17e^{-14}$<br>$4.87e^{-9}$<br>$4.34e^{-6}$	5LY8_A (Distal Tail protein (gp16) from bacteriophage infection, *Lactobacillus casei*, fluorescence; 1.28A [*Lactobacillus* phage J-1]) 100/$2.9e^{-34}$	Phage distal tail protein (Dit)

22	19430/24592	+	Phage minor structural protein	0.0	put_anti_recept (29-352)	$7.89e^{-62}$	1) 3GS9_A (protein gp18 from *Listeria* phage A118) 99.91/$2.7e^{-22}$ 2) 3CDD_B (prophage MuSo2 gpP) 97.99/e^{-8} 3) 4LIN_E gp26 (phage P22) 90.7/0.68 4) COG3501 VgrG (type VI secretion system) 87.03/180 5) 5E7T_B Baseplate protein Bppu (phage Tuc2009) 84.65/14	Phage baseplate hub protein + central tail fiber protein
					Prophage_tail (40-359)	$1.61e^{-15}$		
					Sms (362-641)	$1.73e^{-9}$		
					Myosin_tail_1 (359-627)	$4e^{-7}$		
					SMS_prok_A (362-613)	$5.19e^{-7}$		
					PRK03918 (474-633)	$2.83e^{-3}$		
23	24609/24986	+	hp				1) Stage V sporulation protein T; [*Bacillus subtilis*] 98.15/ $4.4e^{-8}$ 2) Transition state regulatory protein abrB/DNA; AbrB, abrB8, [*Bacillus subtilis*] 98.14 $1.4e^{-7}$	hp
24	25023/25538	+	MULTISPECIES: holin [*Bacillus cereus* group]	0.0	Phage_holin_4_1 (45-131)	$4.81e^{-9}$		Phage holin
					Holin_tox_secr (47-131)	$1.13e^{-5}$		
					COG4824 (50-131)	$5.32e^{-3}$		

25	25519/25728	+	Holin	$3e^{-11}$			1) TIGR01598 (holin_phiLC3) $99.81/1.1e^{-22}$<br>2) PF04531.13 (Phage_holin_1) $99.78/1.8e^{-21}$	Phage holin
26	25806/26636	+	MULTISPECIES: N-acetylmuramoyl-L-alanine amidase [*Bacillus cereus* group]	0.0	GH_PlyB-like (3-176)	$2.54e^{-84}$		Phage lysin, N-acetylmuramoyl-L-alanine amidase
					Acm (4-174)	$9.42e^{-27}$		
					Glyco_hydro_25 (5-168)	$6.8e^{-23}$		
					Amidase02_C (229-273)	e^{-10}		
27	27213/26839	-	hp					hp
28	27613/27425	-	hp				TIGR02978 (phageshock_pspC) 63.63/37	hp
29	28195-27656	-	hp		phage_Gp111 (8-82)	$9.2e^{-6}$		hp
30	28450/28211	-	hp				TIGR02978 (phageshock_pspC) 80.27/7.1	hp
31	28887/28540	-	hp				1) 5CEG_C Addiction module antidote protein, CopG/Arc/MetJ 97.79/0.0000042<br>2) PF09386.10 (ParD) $98.18/2e^{-7}$	Putative ParG-like protein

							3) PF09274.10 ParG ; 97.27/0.000029 4) 1BAZ_A (arc repressor phage P22) 92.02/0.89	
32	30141/28900	-	MULTISPECIES: ParM/StbA family protein [*Bacillus*]	0.0	ParM_like (15-376)	2.35e^{-23}		ParM-family protein
33	31268/30306	-	Site-specific integrase [*Bacillus thuringiensis*]	0.0	DNA_BRE_C (115-302)	4.24e^{-13}		Phage tyrosine based site-specific recombinase
					XerD (4-295)	3.34e^{-9}		
					recomb_XerD (9-295)	1.21e^{-7}		
					Phage_integrase (143-302)	1.64e^{-5}		
					XerC (4-301)	6.71e^{-5}		

34	31912/31358	-	MerR family transcriptional regulator [*Bacillus sp.*]	$4e^{-13}$			1) 3GP4_B (MerR-family transcriptional regulator) $99.42/1.2e^{-13}$ 2) 5I41_B (chromosome-anchoring protein RacA B. subtilis) $98.02/5.3e^{-7}$ 3) 4J2N_B (gp37 Xis-like, Mycobacteriophage Pucovnik) $98.02/4e^{-7}$	hp
35	32522/32244	-	YccF domain-containing protein [*Bacillus thuringiensis*]	$2e^{-61}$	YccF (21-64)	$8e^{-5}$	1) B7GIP6 (Metal-binding Membrane-associated protein OS *Anoxybacillus flavithermus*) $98.15/1.3e^{-8}$	hp
					PRK11770 (33-64)	$9.04e^{-4}$		
36	33914/32646	-	Replication initiator protein A	$3e^{-27}$	RepA_N (20-115)	$8.02e^{-9}$	1) 4PT7_B (replication initiator A family protein) $99.15/3e^{-12}$ 2) PF03428.13 (Replication protein C) 97.33/0.00013	RepA-like replication initiator protein

							3) PF04492.13 (Phage_rep_ O) 97.32/0.00006	
37	35083/34844	-	XRE family transcriptional regulator	$4e^{-12}$	HTH_26 (8-57)	$3e^{-3}$	1) 1ADR_A P22 C2 REPRESSOR {Enterobacteria phage P22} 98.93/$1.1e^{-10}$ 2) PF07022.13 (Phage_CI_repr) 98.49/$1.8e^{-8}$ 2) 2XCJ_B C PROTEIN; [*Enterobacteria* phage P2] 98.78/$4e^{-10}$ 3) 3BD1_A Cro protein; transcription factor, helix-turn-helix, [*Xylella fastidiosa*] 98.54/ $1.8e^{-8}$	Phage XRE family transcriptional repressor
38	36414/35236	+	XRE family transcriptional regulator [*Bacillus thuringiensis*]	$3e^{-177}$			1) 5ZW_B AimR transcriptional regulator; dimer, phage phi3T, 99.93/$8.4e^{-24}$ 2) 3U3W_B (transcriptional activator PlcR) 99.92/$3.7e^{-24}$ 3) AimR transcriptional	AimR-like protein

							regulator, [*Bacillus* phage SPbeta] $99.9/2.9e^{-21}$	
39	36520/36660	+	hp				1) PF05968.11 (Bacillus_PapR) 95.85/0.0049	AimP-like Protein
	36783/36944	+	hp					hp
41	37398/36964	-	XRE family transcriptional regulator [*Bacillus thuringiensis*]	0.0	HTH_XRE (36-92)	$6.54e^{-12}$	1) 3B7H_A (Prophage Lp1 protein 11) $98.69/3.2e^{-9}$ 2) PF07022.13 (Phage_CI_repr) $98.53/7.3e^{-9}$ 3) 2FJR_B (repressor protein CI, phage 186) $98.47/84e^{-9}$ 4) 2CRO_A (cro protein, phage 434) $98.17/2.1e^{-7}$	XRE family transcriptional repressor
					HTH_XRE (37-92)	$1.17e^{-10}$		
					HTH_3 (38-92)	$1.44e^{-9}$		
					HipB (34-110)	$1.06e^{-7}$		
					PRK09706 (34-92)	$1.95e^{-3}$		
42	37591/37791	+	XRE family transcriptional regulator [*Bacillus*]	0.0	HTH_3 (10-64)	$1.44e^{-7}$	1) P22 C2 REPRESSOR $99.44/3.8e^{-14}$	XRE family transcriptional repressor
					HTH_XRE (10-64)	$2.48e^{-7}$		
					HTH_XRE (10-64)	$5.48e^{-7}$		
					XRE (1-61)	$3.68e^{-4}$		

43	37843/37980	+	hp					hp
44	38153/38680	+	Holliday junction resolvase RecU [*B. thuringiensis*]	$4e^{-129}$	recU	$3.1e^{-34}$		Holliday junction resolvase
45	38692/39201	+	hp					hp
46	39223/39678	+	AbrB/MazE/SpoVT family DNA-binding domain-containing protein	e^{-93}	spore_V_T (43-180)	$3.6e^{-11}$		AbrB/MazE/SpoVT family DNA-binding domain-containing protein
					spore_V_T (190-324)	$9.28e^{-8}$		
					AbrB (205-435)	$5.7e^{-7}$		
					AbrB (40-261)	$1.1e^{-6}$		
					MazE_antitoxin (79-177)	$6.3e^{-4}$		
47	39703/40080	+	cell division protein SepF [*B. thuringiensis*]	$3e^{-84}$				Cell division protein SepF [*B. thuringiensis*]
48	40098/40871	+	hp					hp
49	40998/41516	+	hp				1) PF07768.11 (PVL ORF-50-like family) 97.46/0.00002	hp
50	41588/41875	+	hp					hp

51	41872/42099	+	hp					hp
52	42146/42883	+	hp					hp
53	42900/43154	+	hp					hp
54	43151/43321	+	hp					hp
55	43338/43592	+	hp					hp
56	43632/43784	+	hp				ECOD_001108148_e 4mo1A1 (DnaJ/Hsp40 cysteine-rich donain, F: Antiterm_like) 95.9/0.0018 2) TIGR02642 (phage_xxxx) 91.88/0.011 3) KOG2813 (chaperone, DnaJ domain) 86.38/0.12 4) 4MO1_A (antitermination protein Q) 86.29/0.095	DnaJ-domain-containing protein
57	43826/44029	+	hp					hp
58	44061/44267	+	hp					hp
59	44270/44503	+	hp					hp

60	44542/44799	+	hp					hp
61	44799/45203	+	hp					hp
62	45237/45413	+	hp				YflT ; Heat induced stress protein YflT 80.48/4.5	hp
63	45456/45581	+	hp					hp
64	45607/45888	+	hp				1) PF11198.8 (DUF2857) 94.5/0.13	hp
65	45927/46076	+	hp					hp
66	46100/46606	+	phage ArpU family transcriptional regulator	$4e^{-110}$	phage_arpU (64-453)	$2.31e^{-16}$		Phage ArpU family transcriptional regulator
67	47529/47762	+	Transglycosylase [*Bacillus* phage phiCM3]	$3e^{-21}$				hp
68	47782/49002	+	DNA modification methylase [*Bacillus thuringiensis*]	0.0	ParB_N_like_MT (37-291)	$2{,}79e^{-32}$		DNA modification methylase
					YhdJ (496-1188)	$1{,}85e^{-13}$		
					N6_N4_Mtase (595-1167)	$6{,}3e^{-12}$		
					PRK13699 (562-1125)	$3{,}8e^{-9}$		
69	49140/49373	+	hp					hp

70	49389/49646	+	hp				1) Antitermination protein Q 96.65/0.00014<br>2) DnaJ_CXXCXGXG ; DnaJ central domain 95.69/0.0019	DnaJ-domain containing protein
71	49660/49893	+	hp					hp

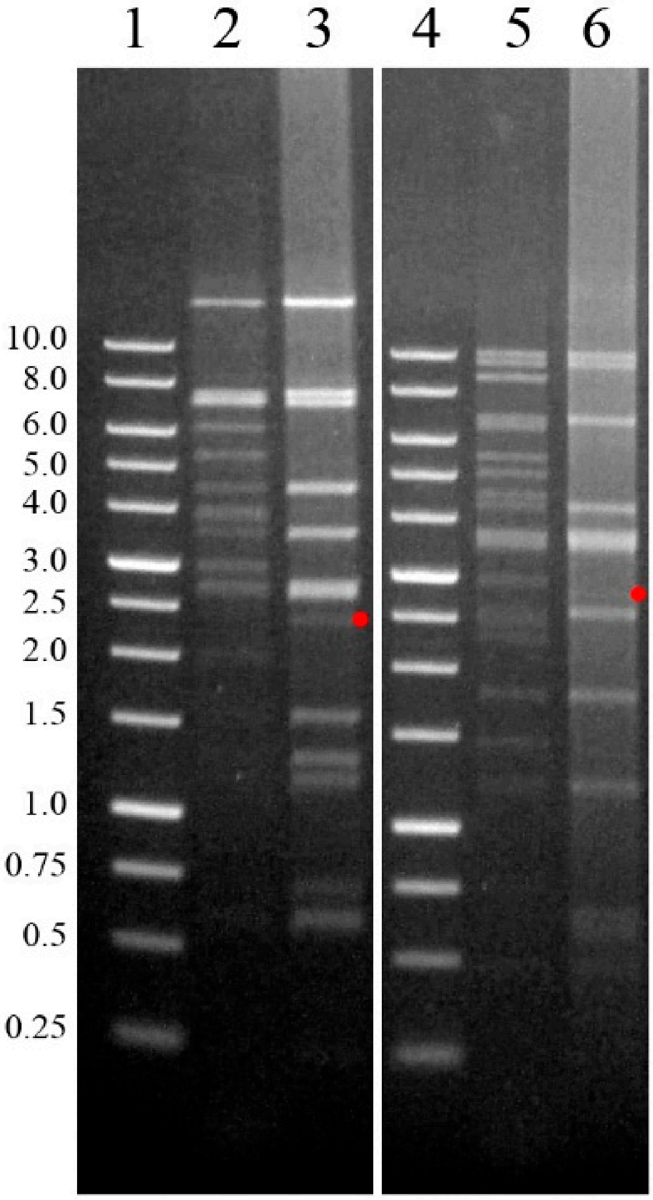

Figure S2: Phage DNA restriction patterns compared with those of the plasmid DNA purified by alkaline lysis method from the host lysogen *B. thuringiensis* VKM B-83. Lanes 1,4 - Molecular weight markers; 2,5 – plasmid DNA digested with HindIII and EcoRI, respectively; 3,6 – phage DNA, digested with HindIII and EcoRI, respectively. *pac*-fragmets (indicated with the red circles) are absent in the lanes 2 and 5, indicating that B83 DNA is indeed circular in the host cytoplasm.

Table S3: Phage genomes used for homologous gene clustering

No.	Name	Genome Accession number
1	*Bacillus* phage vB_BtS_B83	MK759918.1
2	*Bacillus thuringiensis* strain L-7601 plasmid unnamed2	CP020004.1
3	*Bacillus thuringiensis* serovar *indiana* strain HD521 plasmid pBTHD521-2	CP010108.1
4	*Bacillus* phage vB_BtS_BMBtp14	KX190833.1
5	*Listeria* phage PSU-VKH-LP041	MH341453.1
6	*Listeria* phage B054	NC_009813.1
7	*Enterococcus* phage phiFL3B	GQ478087.1
8	*Enterococcus* phage phiFL1C	GQ478083.1
9	*Enterococcus* phage phiFL1B	GQ478082.1
10	*Brevibacillus* phage Emery	KC595516.1
11	Phage 5P_2	MK113950.1
12	*Gordonia* phage Pollux	MH513979.1
13	*Gordonia* phage Bowser	NC_030930.1
14	*Clostridium* phage CDKM15	KX228400.1
15	*Enterococcus* phage phiEf11	NC_013696.1
16	*Clostridium* phage phiMMP02	NC_019421.1
17	*Clostridium* phage phiCD505	NC_028764.1
18	*Clostridium* phage phiCD27	NC_011398.1
19	*Bacillus* phage PBP180	KC847113.1
20	*Lactococcus* phage 28201	NC_031013.1
21	*Lactococcus* phage 98201	NC_031064.1
22	*Rhizobium* phage vB_RleS_L338C	NC_023502.1
23	*Clostridium* phage CP3	KY206887.1
24	*Vibrio* phage 1	JF713456.1
25	*Vibrio* phage Ares1	MG720309.1
26	*Stenotrophomonas* phage vB_SmaS_DLP_5	MG189906.1
27	*Bacillus* phage PfEFR-5	NC_031055.1
28	*Bacillus* phage Carmel_SA	KY963371.1
29	*Bacillus* phage phi4I1	KT967075.1
30	*Bacillus* phage phIS3501	NC_019502.1
31	*Bacillus* phage Tavor_SA	KY963369.1
32	*Bacillus* phage Negev_SA	KY963370.1
33	*Bacillus* phage Gamma	NC_007458.1
34	*Bacillus* phage AP631	MK085976.1
35	*Staphylococcus* phage SpaA1	NC_018277.1
36	*Bacillus* phage phiS58	KT970646.1
37	*Bacillus* phage Waukesha92	NC_025424.1
38	*Bacillus* phage vB_BtS_BMBtp3	NC_028748.2
39	*Bacillus* phage phi4J1	NC_029008.1
40	*Bacillus* phage phi4B1	NC_028886.1
41	*Bacillus* phage BtCS33	NC_018085.1
42	*Bacillus* phage vB_BtS_BMBtp13	KX190832.1
43	*Bacillus* phage 11143	GU233956.1
44	*Bacillus* prophage phBC6A52	NC_004821.1
45	*Bacillus* phage DK1	MK284526.1
46	*Bacillus* phage Stitch	NC_031032.1

47	*Bacillus* phage Aurora	NC_031121.1
48	*Bacillus* phage 250	NC_029024.1
49	*Bacillus* phage IEBH	NC_011167.1
50	*Clostridioides* phage LIBA6276	MF547662.1
51	*Streptococcus* phage D4276	MF161328.1
52	*Streptococcus* phage P4761	KY705258.1
53	*Streptococcus* virus 9872	NC_031094.1
55	*Bacillus* prophage phBC6A51	NC_004820.1
56	*Bacillus* phage PfNC7401	KX227758.1
57	*Bacillus* phage BVE2	MG584725.1
58	*Bacillus* phage vB_BtS_BMBtp16	KT372714.1
60	*Lactobacillus* phage phi jlb1	NC_024206.1
61	*Lactobacillus* phage iLp84	NC_028783.1
62	*Brevibacillus* phage Abouo	NC_029029.1
63	*Streptococcus* phage TP-J34	NC_020197.1
64	*Bacillus* phage BM5	KT995479.1
65	*Bacillus* virus 1	NC_009737.2
66	*Aeribacillus* phage AP45	KX965989.1
67	*Bacillus* phage BMBtp1	KT852578.1
68	*Brevibacillus* phage Davies	NC_022980.1
69	*Exiguobacterium* phage vB_EalM-137	MH884510.1
70	*Geobacillus* phage TP-84	KY565347.2
71	*Bacillus* phage vB_BpsM-61	MH884514.1
72	*Staphylococcus* phage StB27	NC_019914.1
73	*Paenibacillus* phage PG1	NC_021558.1
74	*Bacillus* phage Ray17	MH752385.1
75	*Paenibacillus* phage Dragolir	MG727697.1
76	*Paenibacillus* phage Vegas	NC_028767.1
77	*Paenibacillus* phage Wanderer	MH431930.1
78	*Bacillus* phage BCD7	NC_019515.1
79	*Bacillus* phage TP21-L	NC_011645.1
80	*Bacillus* phage BMBtpLA3	KX190834.1
82	*Bacillus* phage Deep-Purple	MF176161.1
83	*Bacillus* phage TsarBomba	NC_028890.1
84	*Bacillus* phage BCP78	NC_018860.1
85	*Bacillus* phage QCM8	KX961630.1
86	*Bacillus* phage BC01	MH487649.1
87	*Bacillus* phage PBC6	KT187252.1
88	*Bacillus* phage Bcp1	NC_024137.1
89	*Bacillus* phage PBC5	KT070868.1
90	*Bacillus* phage Mater	NC_027366.1
91	*Bacillus* phage Eldridge	NC_030920.1
92	*Bacillus* phage Belinda	NC_031024.1
93	*Bacillus* phage Spock	NC_022763.1
94	*Bacillus* phage phiAGATE	NC_020081.2
95	*Bacillus* phage Bp8p-C	NC_029121.1
96	*Bacillus* phage Bobb	NC_024792.1
97	*Pseudomonas* phage phiPsa374	NC_023601.1
98	*Thermus* phage phi OH2	NC_021784.1
99	*Erysipelothrix* phage phi1605	MF172979.1

100	*Streptococcus* phage phi1207.3	AY657002.1
101	*Faecalibacterium* phage FP_Epona	MG711462.1
102	*Streptococcus* phage phiSC070807	KT336321.1
103	*Streptococcus* phage phi-SsUD.1	FN997652.1
104	*Fusobacterium* phage Funu1	KR131710.1
105	*Acidithiobacillus* phage AcaML1	JX507079.1
106	*Gordonia* phage Danyall	MH479910.1

Table S4: Phage genomes used for phylogenetic inference

No	Name	Genome Accession number	Number of homologous genes (with respect to B83)	ORF number in B83 genome	TerL Accession Number
1	*Bacillus* phage vB_BtS_B83	MK759918.1			QCQ57785.1
2	*Bacillus thuringiensis* L-7601 plasmid2	CP020004.1	71	1-71	AQY42398.1
3	*Bacillus thuringiensis* serovar *indiana* strain HD521 plasmid pBTHD521-2	CP010108.1	40	1, 2, 5, 6, 9, 10, 11, 12, 13, 14, 15, 16, 17, 18, 19, 21, 22, 23, 27, 29, 30, 33, 35, 37, 38, 39, 40, 41, 45, 46, 47, 48, 63, 64, 65, 66, 67, 69, 70, 71	AKR38528.1
4	*Bacillus* phage vB_BtS_BMBtp14	KX190833.1	34	1, 2, 4, 5, 6, 9, 10, 11, 12, 13, 14, 15, 16, 17, 18, 21, 29, 30, 33, 34, 37, 38, 41, 42, 43, 44, 45, 46, 47, 48, 59, 64, 66, 69	ANT40015.1
5	*Clostridium* phage Cp3	MF001357.1	6	4, 5, 6, 9, 10, 12	ASZ76605.1
6	*Clostridium* phage Clo-PEP-1	KY206887.1	6	4, 5, 6, 9, 10,12	APQ41975.1
7	*Bacillus* phage vB_BtS_BMBtp3	NC_028748.2	6	2, 21, 38, 44, 47, 62	YP_009193979.1
8	*Bacillus* phage IEBH	NC_011167.1	4	21, 27, 38, 57	YP_002154374.1
9	*Bacillus* phage Waukesha92	NC_025424.1	4	21, 38, 44, 47	YP_009099334.1
10	*Bacillus* phage PBP180	KC847113.1	4	5, 9, 10, 12	AGK88023.1
11	*Bacillus* phage phi4I1	KT967075.1	4	21, 26, 66, 67	ALN97345.1
12	*Bacillus* phage BtCS33	NC_018085.1	4	21, 26, 66, 67	YP_006488672.1
13	*Bacillus* phage phIS3501	NC_019502.1	4	21, 26, 66, 67	YP_007004362.1
14	*Bacillus* phage Carmel_SA	KY963371.1	4	21, 37, 38 67	ARW58516.1
